# Sliding mode controller–observer pair for p53 pathway

**DOI:** 10.1049/iet-syb.2018.5121

**Published:** 2019-07-01

**Authors:** Muhammad Rizwan Azam, Vadim I. Utkin, Ali Arshad Uppal, Aamer Iqbal Bhatti

**Affiliations:** ^1^ CASPR Department of Electronics Engineering Capital University of Science & Technology Islamabad Pakistan; ^2^ Electrical and Computer Engineering Department The Ohio State University Columbus Ohio USA; ^3^ Department of Electrical and Computer Engineering COMSATS University Islamabad Pakistan

**Keywords:** control system synthesis, tumours, variable structure systems, observers, cancer, robust control, drugs, proteins, medical control systems, wild‐type p53, nonlinear technique, drug design, control‐oriented p53 model, control input, drug infusion, smooth control signal, dynamic SMC, zero‐dynamics, model‐based control design, input disturbance, controlled drug administration, sliding mode controller–observer pair, cancerous cells, antitumour agent, molecule based drug

## Abstract

A significant loss of p53 protein, an anti‐tumour agent, is observed in early cancerous cells. Induction of small molecules based drug is by far the most prominent technique to revive and maintain wild‐type p53 to the desired level. In this study, a sliding mode control (SMC) based robust non‐linear technique is presented for the drug design of a control‐oriented p53 model. The control input generated by conventional SMC is discontinuous; however, depending on the physical nature of the system, drug infusion needs to be continuous. Therefore, to obtain a smooth control signal, a dynamic SMC (DSMC) is designed. Moreover, the boundedness of the zero‐dynamics is also proved. To make the model‐based control design possible, the unknown states of the system are estimated using an equivalent control based, reduced‐order sliding mode observer. The robustness of the proposed technique is assessed by introducing input disturbance and parametric uncertainty in the system. The effectiveness of the proposed control scheme is witnessed by performing *in‐silico* trials, revealing that the sustained level of p53 can be achieved by controlled drug administration. Moreover, a comparative quantitative analysis shows that both controllers yield similar performance. However, DSMC consumes less control energy.

## 1 Introduction

Cancer remains one of the leading causes of death in the human race, which mainly develops as a consequence of oncogenes activation and inactivation of tumour suppressors. In recent years, tumour suppressor protein: p53 has become a mainstream target in anti‐tumour drug development [1]. After the discovery of the p53 protein in 1979, the scientists have invested a considerable amount of effort in exploring the protein. It has been observed that around 50% of cancer cases contain either mutations or inactivation of the p53 protein. The protein attains the significance due to its role in cancer suppression and its ability to respond to various stresses which are toxic for the genome. In its wild‐type state, p53 induces responses like DNA repair mechanism, senescence, cell cycle arrest, and cell death [2]. Whenever the cell gets endangered by stresses (e.g. radioactivity or DNA damage), p53 activates multiple downstream targets to ensure normal functioning of the cell. In fact, whenever the genome's integrity is questioned, p53 plays its role to preserve it, hence named ‘guardian of the genome’ [3].

The critical role of p53 in regulating numerous cellular processes demands precise control of its level and activity. Under normal circumstances, the concentration of p53 is maintained at a low steady‐state level. The murine double minute 2 (MDM2) protein is the primary negative regulator of p53, which serves as it's E3 ligase [4]. p53 and MDM2 constitute an auto‐regulatory feedback loop for mutual regulation. MDM2 is responsible for the destruction of p53 through the ubiquitination process, while the activation of p53 causes transcription of MDM2 mRNA, which in turn increases the level of MDM2 protein [4, 5].

The MDM2 attaches a phosphate ion with p53 to initiate its degradation by the proteasome [6]. In many tumours, overexpression of MDM2 is the reason for reduced levels of p53, which prevents DNA damage repair, cell cycle arrest, and apoptosis. Thus, inhibiting the protein–protein interaction between p53 and MDM2 can activate and restore the levels of wild‐type p53, which in turn, can restore the normal cell functionality through p53 mediated responses [7]. Hence, due to the same reason, MDM2 is becoming a mainstream therapeutic target in the cancerous cells [8, 9].

The p53 protein binds with MDM2 through hydrophobic residues at designated binding pockets [4]. It is revealed from the structure of p53 that some small non‐peptide molecules can mimic the binding pattern between p53 and MDM2. These molecules can prevent the protein–protein interaction amongst p53 and MDM2 leading to increased accumulation of p53. Blocking the protein–protein interaction through such molecule inhibitors are emerging as a promising therapeutic strategy for human cancer retaining wild‐type p53 [10]. Numerous small molecule inhibitors have been reported in recent years, many of which have already completed successful preclinical and clinical trials. Nutlin is a family of such molecule inhibitors, which binds to MDM2 with a higher affinity, without creating genotoxicity. Nutlin binds to N‐terminal pocket of MDM2, precisely where p53 binds [8, 11]. Nutlin‐3a is reported to have restored wild‐type p53 functionality, while some other variants of Nutlin have effectively treated tumours with dysfunctional or mutant p53 [12].

The complex feedback interactions of the p53 pathway govern its dynamic response. Initial studies were aimed at measuring the dynamics at the cell population level [13]. However, later on, it was realised that measuring the dynamics in the population may hide the actual behaviour expressed by single cells. Hence analysing the fluorescence‐tagged protein reveals the hidden dynamics of an individual cell [14]. Depending on the stimulus, the p53–MDM2 loop can exhibit multiple dynamic response patterns. Broadly, these patterns are either oscillatory or sustained [15]. p53 is reported to initiate oscillations in case of less extensive DNA damage. The oscillatory behaviour is further classified as digital pulses, damped oscillations, and sustained oscillations. The frequency of these oscillatory pulses is dependent upon the extent of the DNA damage, while the pulse width and amplitude are invariant. The pulsating p53 is usually associated with DNA repair or cell cycle arrest [12, 14]. The status of the DNA is verified after each pulse of ∼6 h. In the case the DNA is repaired, the oscillatory p53 dies out and resumes the blocked cell cycle process. The sustained p53 response is initiated due to extensive DNA damage. The amplitude and width of the response are directly dependent upon the extent of the damage. The expressed genes, in this case, lead to the irreversible cell fate, i.e. cell death [16, 17]. It is evident that in the case of severe DNA damage, p53 does not provide adequate time for DNA to repair, and kills the cell immediately.

The computational frameworks provide useful tools to better understand the network topology, create a new hypothesis and explore the areas for which we lack complete understanding. The efforts to model the p53 pathway are mainly focused upon the interactions between P53 and MDM2 governing its responses [18]. Numerous mathematical models have been developed in the literature using continuous time, discrete time and delayed differential equations [19, 20, 21]. ‘Systems biology’ has long been used to understand and to predict the behaviour of biological systems through computational models. Recently, systems biology, along with the control theory, have been considered as a great tool for a more precise therapeutic intervention in complex biological networks. Nevertheless, some noteworthy developments have been made in drug delivery of cardiovascular systems [22, 23], blood pressure control [24, 25], tumour chemotherapy [26], anaesthesia drug delivery [27], diabetes control [28], Parkinson's tremor [29] and HIV/AIDS control [30, 31].

Application of control in cancer treatment is a fairly new subject. The main objective in cancer treatment is remission of cancerous cells within minimum time while maintaining the health profile of a patient. Chemotherapy, radiotherapy and surgical procedures are one way around, but these procedures may reduce the quality of life of the patient [32]. The current research trend is shifting towards in‐silico methods for analysis and control. There is a strong need to use these in‐silico models to implement drug design using control theory. The control objective is to administer the treatment and to schedule the drug. The mathematical models of bio‐systems are not always precise. Therefore, it is required to solve the desired task by designing a control system, which can handle the model imprecisions and uncertainties. In the literature, a couple of model‐based control techniques are explored for the p53 pathway. In [33], a complex mathematical model for the p53 and related pathways is exploited to design flatness based control for maintaining the desired level of p53. In our previous work [34], we designed a Lyapunov‐based control system to obtain the desired model concentrations by shifting the equilibrium point from cancerous to normal state. Therein, all the state variables are considered to be measurable, which is not the case in actual scenarios.

Both of the above‐mentioned control techniques are not inherently robust. The issue of robustness is addressed in this paper by the application of sliding mode control (SMC), which is known for its robustness properties [35]. The main issues accompanied by SMC, i.e. chattering and discontinuous control input are addressed by employing a modified algorithm based upon the theory of dynamic SMC (DSMC). As the discontinuous term is included in the time derivative of the control input, therefore, the output of DSMC is smooth [36]. To make model‐based control possible, a reduce‐order sliding mode observer (SMO) is developed to estimate the unknown states. The robustness of the proposed scheme is assessed by introducing parametric uncertainties, measurement noise, and an input disturbance. The loss in the concentration of drug Nutlin due to unwanted cross‐talk between pathways or due to undesirable signals from neighbouring cells is considered as an input disturbance. Moreover, a quantitative comparison is also made between the DSMC and the conventional SMC, which shows that the DSMC consumes the lesser control energy for similar tracking performance.

The structure of the paper is organised as follows. In Section 2, ordinary differential equations (ODEs)‐based control‐oriented non‐linear mathematical model of the p53 pathway is discussed. The designs of conventional and dynamic SMC algorithms along with the corresponding SMOs are discussed in Sections 3 and 4, respectively. The results and discussions are presented in Section 5 and finally, the paper is concluded in Section 6.

## 2 Mathematical model

**Fig. 1:**
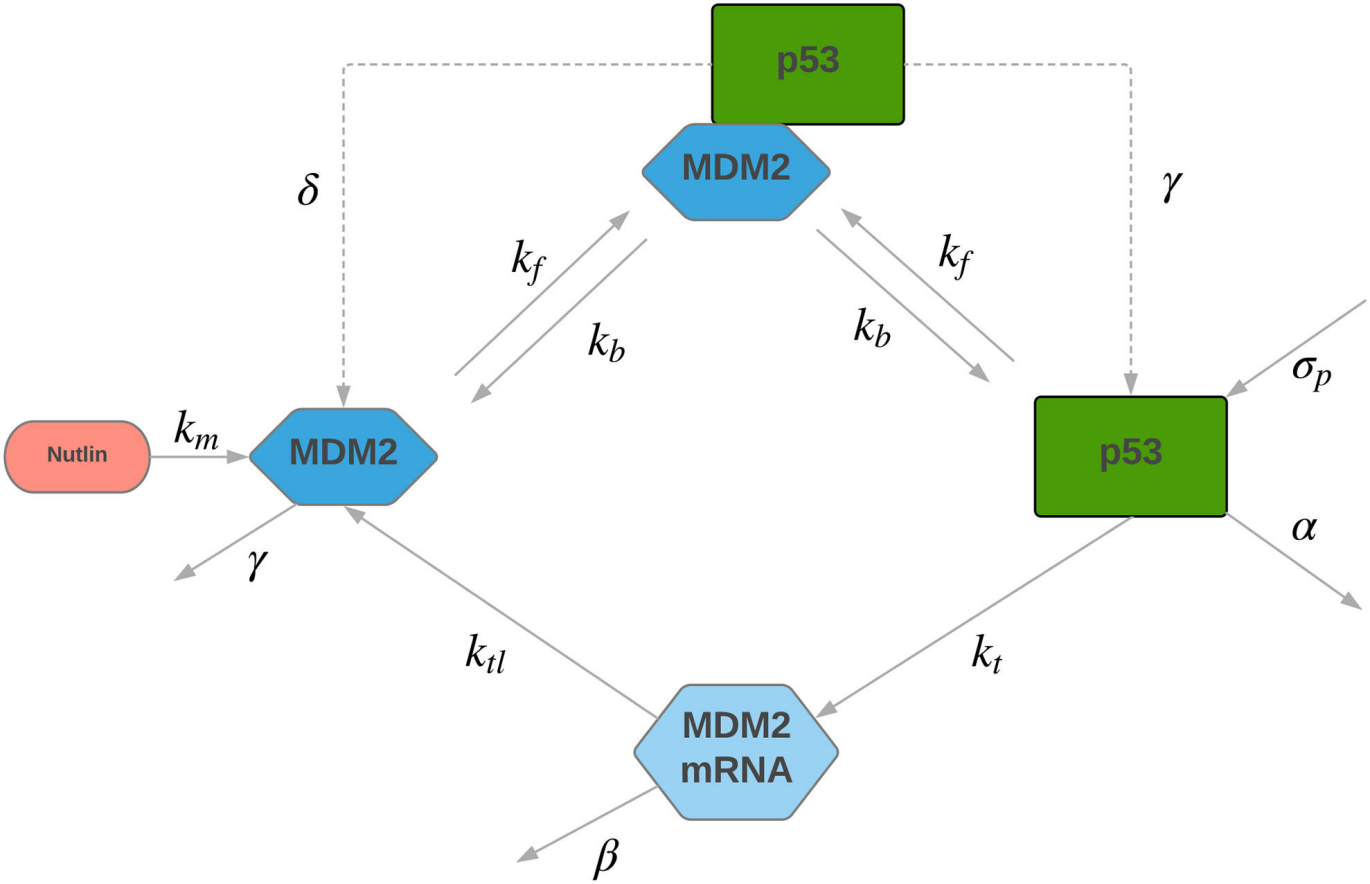
Schematic model of p53 pathway dynamics

The mathematical model presented by Hunziker *et al.* in [21] allows control‐oriented drug dosage design. The model offers a simplistic approach yet adequately preserves the fundamental dynamical properties of the p53–MDM loop. The interactions between MDM2 and p53 protein are represented by a schematic diagram shown in Fig. 1 [21]. The single cellular dynamics of the pathway are demonstrated by an ODE‐based mathematical model, given by

(1) $$\begin{array}{rl} {\dot{x}}_{1} & =\sigma_{p}-\alpha x_{1}-k_{f}x_{1}x_{3}+k_{b}x_{4}+\gamma x_{4},\\ {\dot{x}}_{2} & =k_{t}x_{1}^{2}-\beta x_{2},\\ {\dot{x}}_{3} & =k_{tl}x_{2}-k_{f}x_{1}x_{3}+k_{b}x_{4}+\delta x_{4}-\gamma x_{3}-k_{m}(u-\zeta )x_{3},\\ {\dot{x}}_{4} & =k_{f}x_{1}x_{3}-k_{b}x_{4}-\delta x_{4}-\gamma x_{4}. \end{array}$$

where $x_{1}$ is the concentration of p53 protein, $x_{2}$ is the Mdm2 mRNA, $x_{3}$ is the concentration of MDM2 protein and $x_{4}$ is the MDM2–p53 protein complex. All of these concentrations are measured in nM. The control input *u* to the system is the concentration of the anti‐tumour drug ‘Nutlin’, measured in mg/kg (Note: $x_{3}$ is positive by physical nature, and takes part as control gain) and the concerned output is $x_{1}$ (concentration of p53 protein). Here, ζ is the input disturbance, faced by cellular structure due to intrinsic noise, unwanted interference from neighbouring pathways and environmental stresses. It appears with the same vector g as the input *u*, hence ζ is assumed to be a matched disturbance. The disturbance satisfies the following assumption:

Consider ζ to be a matched disturbance (bounded by $∥\zeta ∥\leq \zeta_{0}$ and $\zeta_{0}\in R^{+}$), which is sufficiently smooth, i.e. $\dot{\zeta }$ is the continuous and bounded, i.e. $\dot{\zeta }(t)\leq \psi (t),∥\psi (t)∥\leq \psi_{0}$, where ψ(t) is a smooth function and $\psi_{0}\in R^{+}$.

The parameters and rate constants being used in the p53 model are listed and described in Table 1. Here, the Greek letters (α, β, γ and δ) represent the degradation rates. The parameter α models all the processes which result in Mdm2 independent deactivation of the p53 protein, leading to a reduced active p53 concentration in the nucleus. Whereas the parameter δ represents the Mdm2 dependent p53 deactivation. The parameter β is the degradation rate of Mdm2 mRNA and γ is the Mdm2 protein degradation, due to the auto‐ubiquitination process.

**Table 1:** Definition of model parameters and kinetic rate constants [21]

Parameter	Definition	Value
$\sigma_{p}$	production rate of p53	1000 nMh^−1^
α	Mdm2 independent deactivation/degradation of p53	0.1 h^−1^
δ	Mdm2 dependent deactivation/degradation of p53	11 h^−1^
$k_{t}$	transcription of Mdm2	0.03 (nMh)^−1^
$k_{t}l$	translation of Mdm2	1.4 h^−1^
β	degradation rate of Mdm2 mRNA	0.6 h^−1^
γ	Mdm2 degradation/deactivation	0.2 h^−1^
$k_{b}$	dissociation of Mdm2–p53	7.2 h^−1^
$k_{m}$	Nutlin rate constant	200 h^−1^
$k_{D}=k_{b}/k_{f}$	dissociation constant of Mdm2–p53	1.44 nM

The subscripted letters represent the production rates, such as the parameter $\sigma_{p}$ models the synthesis of p53 protein, which is assumed to be produced at a constant rate. The rate constant $k_{t}$ describes the transcription of Mdm2 mRNA, whereas the subsequent translation to Mdm2 protein is described by the rate constant $k_{tl}$. The rate constants $k_{f}$ and $k_{b}$ describe the Mdm2–p53 complex formation and breakup, respectively. Even though most of the parameters are constrained, the parameters γ, δ and $k_{f}$ can vary by the environmental conditions and due to cell–cell variability. The uncertain parameters are listed in Table 2.

**Table 2:** Parameters subjected to variations

Parameter	Nominal value	Actual value	Unit
γ	0.2	0.24	$h^{-1}$
δ	11	13.2	$h^{-1}$
$k_{f}$	5.1428	6.168	$nM^{-1}h^{-1}$

The non‐linear model presented in (1) can be written in control affine form, i.e.

(2) $$\dot{x}=f(x)+g(x)(u+\zeta ),$$

where $x\in R^{4}$ is the state vector, $f,g\in R^{4}$ are smooth vector fields. The vector fields f(x) and g(x) are given as

$$f(x)=\left(\begin{array}{c} \sigma_{p}-\alpha x_{1}-k_{f}x_{1}x_{3}+k_{b}x_{4}+\gamma x_{4}\\ k_{t}x_{1}^{2}-\beta x_{2}\\ k_{tl}x_{2}-k_{f}x_{1}x_{3}+k_{b}x_{4}+\delta x_{4}-\gamma x_{3}\\ k_{f}x_{1}x_{3}-k_{b}x_{4}-\delta x_{4}-\gamma x_{4} \end{array}\right),$$

$$g(x)=\left(\begin{array}{c} 0\\ 0\\ -k_{m}x_{3}\\ 0 \end{array}\right).$$

Recent techniques, such as microscopy, flow cytometry, rapid immunoassay and immunomagnetic‐electrochemiluminescent (ECL) are used for the rapid measurements of p53 and Mdm2 concentrations using patient's serum [37, 38, 39]. Accordingly, the measurement vector $y_{m}$ is given by

(3) $$y_{m}=\begin{array}{cc} {}[x_{1} & x_{3}]{\;}^{T}. \end{array}$$

The objective of this paper is to design a control system to achieve a desired concentration level of p53 in the presence of parameter variations and disturbance. Therefore, the next section illustrates the procedure of control design.

## 3 SMC design

The control objective is to achieve a desired constant level of $x_{1}$, i.e. $x_{1}\to x_{1d}$. The design procedure consists of two steps: in the first step, the variable $x_{3}$ is handled as a fictitious control, represented by a state function $x_{3f}$, defined by

(4) $$x_{3_{f}}=\frac{1}{k_{f}x_{1}}(\sigma_{p}-\alpha x_{1}+(k_{b}+\gamma )x_{4}+k(x_{1}-x_{1d})),$$

substituting $x_{3}=x_{3f}$ in (1), yields

(5) $${\dot{x}}_{1}=-k(x_{1}-x_{1d}).$$

The solution of (5) is given as

(6) $$x_{1}(t)=x_{1d}+(x_{1}(0)-x_{1d})e^{-kt}.$$

For a positive value of *k*, $x_{1}\to x_{1d}$ asymptotically.

The second step employs a selection of real control *u* such that

(7) $$x_{3}=x_{3_{f}}.$$

Therefore, the sliding surface is chosen to be the error between $x_{3}$ and $x_{3_{f}}$ i.e.

(8) $$s=x_{3}-x_{3_{f}},$$

and the control input is chosen to be a discontinuous function

(9) u=Msign(s),M>0.

The problem in (7) is solved should sliding mode occur on s=0.

### 3.1 Existence of sliding mode

The existence of sliding mode can be analysed by taking a positive definite Lyapunov function

(10) $$V=\frac{1}{2}s^{2}>0.$$

The time derivative of the Lyapunov function in (10) is found to be

(11) $$\dot{V}=s\dot{s}.$$

The original system includes parameter variations and external disturbance. To find the stability of the original system, we consider the time derivative of the perturbed sliding variable, that can be found from (2) and (4). Consequently (11) takes the following form:

(12) $$\begin{array}{rl} \dot{V} & =s(\theta (x,t)+\upsilon (x,t)-k_{m}x_{3}M\text{sign}(s)+k_{m}x_{3}\zeta ),\\ \leq & -M{\bar{x}}_{3}k_{m}|s|+|s|\Theta +|s|\Upsilon +|s|{\bar{x}}_{3}k_{m}\zeta_{0},\\ \leq & -|s|(M{\bar{x}}_{3}k_{m}-\Theta -\Upsilon -{\bar{x}}_{3}k_{m}\zeta_{0}). \end{array}$$

where $∥\theta (x,t)∥\leq \Theta \in R^{+}$ contains the nominal model parameters and $∥\upsilon (x,t)∥\leq \Upsilon \in R^{+}$ accommodates the parametric uncertainties. The mathematical expressions for θ and Υ are given as

$$\begin{array}{rl} \theta (x,t) & =(k_{tl}x_{2}-k_{f}x_{1}x_{3}+(k_{b}+\delta )x_{4}-\gamma x_{3})\\ & \;-\left(\frac{1}{x_{1}^{2}}((k_{b}+\gamma )(x_{1}{\dot{x}}_{4}-x_{4}{\dot{x}}_{1})-(\sigma_{p}-kx_{1d}){\dot{x}}_{1})\right), \end{array}$$

$$\begin{array}{rl} \Upsilon (x,t) & =\frac{1}{x_{1}^{2}}\left(\Delta \gamma (x_{1}{\dot{x}}_{4}-x_{4}{\dot{x}}_{1})\right.\\ & \;-\left.\left(\sigma_{p}-kx_{1d})(\Delta \gamma x_{4}-\Delta k_{f}x_{1}x_{3}\right)\right). \end{array}$$

It is pertinent to mention that $x_{3}$ always satisfies the condition $x_{3}>{\bar{x}}_{3}>0$. If the condition $M\geq (\tau +\Theta +\Upsilon +{\bar{x}}_{3}k_{m}\zeta_{0})/({\bar{x}}_{3}k_{m})$ holds, where $\tau \in R^{+}$, then time derivative of Lyapunov function becomes

(13) $$\dot{V}\leq -\sqrt{2V}\tau .$$

The inequality in (13) guarantees that sliding mode $\left(s=0\right)$ is enforced after a finite time interval $t_{s}$ [40], characterised by

(14) $$t_{s}\leq \frac{\sqrt{2Vs(0)}}{\tau }.$$

After the establishment of sliding mode, $x_{3}=x_{3f}$ and eventually $x_{1}=x_{1d}$.

### 3.2 Zero dynamics

It is mandatory to check the stability of zero dynamics after sliding mode has been established. The relative degree *r* of the sliding variable is equal to 1, as *u* appears in $\dot{s}$. Therefore, the system exhibits zero dynamics involving states $x_{2}$, $x_{3}$ and $x_{4}$. Under sliding mode, s=0 $\Rightarrow x_{3}=x_{3f}$, and $x_{1}=x_{1d}$. Now the zero dynamics is governed by

(15) $${\dot{x}}_{2}=k_{t}x_{1d}^{2}-\beta x_{2},$$

(16) $$\begin{array}{rl} {\dot{x}}_{4} & =k_{f}x_{1d}x_{3f}-(k_{b}+\delta +\gamma )x_{4}\\ & =(\sigma_{p}-\alpha x_{1d})-\gamma x_{4}. \end{array}$$

The solutions of the linear ODEs (15) and (16) are given by

(17) $$x_{2}(t)=(x_{2}(0)-\Theta )e^{-\beta t}+\Theta ,$$

(18) $$x_{4}(t)=(x_{4}(0)-\xi )e^{-\gamma t}+\xi ,$$

where $\Theta ,\;\xi \in R^{+}$ are given by

$$\Theta =\frac{k_{t}x_{1d}^{2}}{\beta },$$

$$\xi =\frac{\sigma_{p}-\alpha x_{1d}}{\gamma }.$$

It is obvious from (17) and (18), that $x_{2}$ and $x_{4}$ are bounded.

The control law (9) directly depends upon variables $x_{1},x_{3}$ and $x_{4}$. The measurements of only $x_{1}$ and $x_{3}$ are available. Hence there is a need to design an observer to estimate the unknown state $x_{4}$. It is worth mentioning that we do not need $x_{2}$ in control explicitly, but to enforce sliding mode, $x_{2}$ must be available. Sliding mode existence condition is based on inequality (12). Therefore it is sufficient to know an upper estimate $x_{2\max}$ only. It demonstrates the robustness of sliding mode concerning an unknown state $x_{2}$.

### 3.3 Sliding mode observer

Implementation of the above control requires variable $x_{4}$, which can be found by using a reduced‐order state observer. In this paper, a reduced‐order SMO is proposed to estimate the unknown state. The observer eliminates the need to estimate the state variables which are readily available. Control input *u* (9) is a function of states $x_{1}$, $x_{3}$ and $x_{4}$. The unknown state variable $x_{4}$ can be estimated by enforcing sliding mode on error term ${\tilde{x}}_{1}$, equal to the difference between its real value $x_{1}$ and the estimate ${\hat{x}}_{1}$. The time derivative of ${\hat{x}}_{1}$ is taken as

(19) $${\hat{\dot{x}}}_{1}=\sigma_{p}-\alpha x_{1}-k_{f}x_{1}x_{3}+\mu \text{sign}({\tilde{x}}_{1}).$$

where

(20) $${\tilde{x}}_{1}=x_{1}-{\hat{x}}_{1},$$

and

(21) $${\tilde{\dot{x}}}_{1}={\dot{x}}_{1}-{\hat{\dot{x}}}_{1}=(k_{b}+\gamma )x_{4}-\mu \text{sign}({\tilde{x}}_{1}).$$

The sliding mode with ${\tilde{x}}_{1}=0$ is established if $\mu >|(k_{b}+\gamma )x_{4_{\max}}|$. Then sliding mode equation is defined by equivalent control [35]

$$(\mu \text{sign}({\tilde{x}}_{1})){\;}_{\text{eq}}=(k_{b}+\gamma )x_{4},$$

which can be obtained by a low‐pass filter

(22) $$\begin{array}{rl} \tau \dot{z}+z & =\mu \text{sign}({\tilde{x}}_{1}),\\ \lim_{z\to 0}z & =(\mu \text{sign}({\tilde{x}}_{1})){\;}_{\text{eq}}. \end{array}$$

Eventually $x_{4}$ can be obtained as

(23) $$x_{4}=\frac{z}{\left(k_{b}+\gamma \right)}.$$

**Fig. 2:**
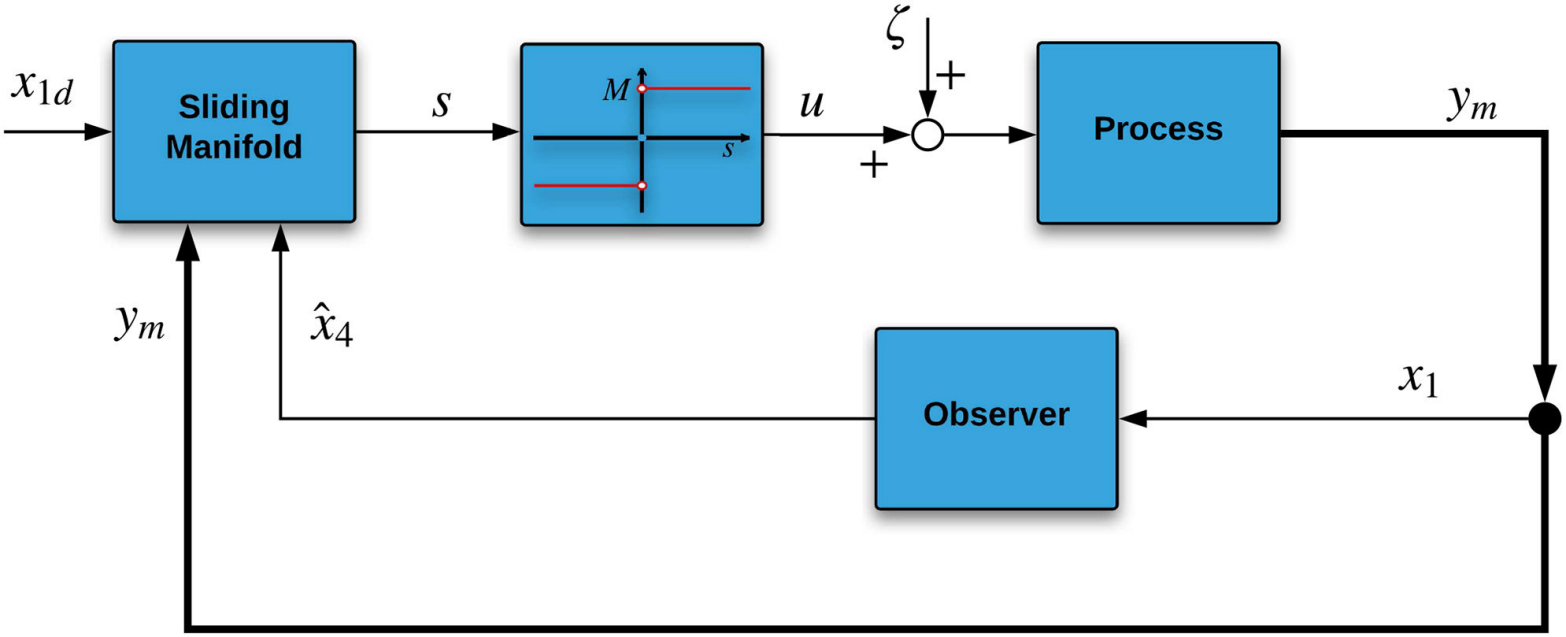
Control implementation scheme‐I

Fig. 2 illustrates the overall implementation scheme of the SMC in conjunction with the reduced‐order SMO. It is worth mentioning that the estimation behaviour of the SMO can be well analysed when we initialise both the p53 plant and SMO with different initial conditions.

Although the discontinuous control *u* in (9) provides robustness against modelling uncertainties, but the modelling imperfections can result in an unwanted high‐frequency motion, called chattering. During this high‐frequency motion, the system is unable to maintain its trajectories on the switching manifold; rather they cross it. The requirement of smoothness in control input and the limitations in actuators for biological control processes limit the application of discontinuous SMC. The inherent properties associated with the SMC (i.e. robustness and parameter invariance) can still be exploited by modifying the discontinuous controller. Hence, in the subsequent section, we discuss the modified control algorithm strategy to obtain a continuous and smooth control input.

## 4 Modified control algorithm

As the control input cannot be discontinuous, so the discontinuous *sign* function is shifted in the time derivative of the control input. The modified technique is inspired by DSMC, which provides a continuous control input along with the inherent properties of SMC. A new sliding variable is proposed, which shifts the discontinuous function (9) into the first‐order time derivative of the control input. The desired trajectory tracking for the output is achieved with the choice of sliding function proposed in (8). A new sliding manifold σ is defined in terms of the sliding manifold *s*, i.e.

(24) $$\sigma =\dot{s}+\lambda s,$$

where *s* is given by (8).

**Fig. 3:**
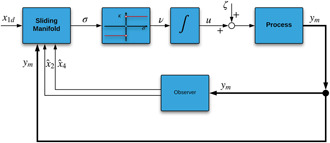
Control implementation scheme‐II

The dynamics of the sliding mode $\left(\sigma =0\right)$ is governed by

(25) $$\dot{s}+\lambda s=0,$$

where λ>0 defines the convergence rate of *s*. This new sliding surface can be considered as a filtered version of *s*, with $\dot{u}=\nu$, where ν=κsign(σ). The complete implementation scheme with the modified controller is presented in Fig. 3.

### 4.1 Existence of sliding mode

The existence of the sliding mode for the modified control design is also analysed by taking a positive definite Lyapunov function

(26) $$V=\frac{1}{2}\sigma^{2}>0.$$

The time derivative of the Lyapunov function (26) is computed as

(27) $$\dot{V}=\sigma \dot{\sigma }.$$

By considering the parametric perturbations and the disturbance, the time derivative of the sliding variable can be found from (2) and (24). Consequently (27) takes the following form:

(28) $$\begin{array}{rl} \dot{V} & =\sigma (\Omega (x,t,u)-k_{m}x_{3}(\dot{u}-\dot{\zeta })+\Psi (x,t)),\\ \dot{V} & \leq -\kappa k_{m}{\bar{x}}_{3}|\sigma |+|\sigma |\Omega_{0}+|\sigma |k_{m}{\bar{x}}_{3}\psi_{0}+|\sigma |\Psi_{0},\\ \dot{V} & \leq -|\sigma |(\kappa k_{m}{\bar{x}}_{3}-\Omega_{0}-k_{m}{\bar{x}}_{3}\psi_{0}-\Psi_{0}). \end{array}$$

where the function $∥\Omega (x,t,u)∥\leq \Omega_{0}\in R^{+}$ contains the nominal model parameters and $∥\Psi (x,t)∥\leq \Psi_{0}\in R^{+}$ accommodates the parametric uncertainties. The sliding mode can be enforced and reachability condition $\left(\dot{V}\leq 0\right)$ can be achieved by selecting a discontinuous controller gain $\kappa \geq (\epsilon +\Omega_{0}+k_{m}{\bar{x}}_{3}\psi_{0}+\Psi_{0})/(k_{m}{\bar{x}}_{3})$, where $\epsilon \in R^{+}$. The time derivative of *V* becomes

(29) $$\dot{V}\leq -\sqrt{2V}\epsilon ,$$

and the system trajectories will converge to the desired state within finite time $t_{s}$, defined by

(30) $$t_{s}\leq \frac{\sqrt{2V\sigma (0)}}{\epsilon }.$$

The new sliding variable σ (24) requires the states $x_{2}$ and $x_{4}$. The estimation of the $x_{4}$ has been discussed in Section 3, whereas the reconstruction of $x_{2}$ is discussed in the subsequent subsection.

### 4.2 Modified SMO

Fig. 3 represents the complete implementation scheme for the modified controller accompanied by the observer. The estimation of $x_{2}$ is carried out similarly as the reconstruction of $x_{4}$ has been performed. Here, the sliding mode is enforced in the manifold ${\tilde{x}}_{3}=x_{3}-{\hat{x}}_{3}$. The structure of the reduced‐order SMO is

(31) $${\hat{\dot{x}}}_{3}=(k_{b}+\delta )x_{4}-k_{f}x_{1}x_{3}-(\gamma +k_{m}u)x_{3}+\vartheta \text{sign}({\tilde{x}}_{3}).$$

It is worth mentioning that $x_{4}$ is used instead of ${\hat{x}}_{4}$ (estimated in Section 3.3) in (31). By selecting a suitable discontinuous gain μ, it has been ensured that $x_{4}$ is already estimated during the estimation of $x_{2}$. From (19), it can be seen that the system trajectories reach the sliding manifold ${\tilde{x}}_{1}=0$ in finite time $ts_{1}$, which is inversely proportional to the discontinuous gain μ [40]. Afterwards, $x_{4}$ is estimated by simply applying a low‐pass filter, as in (23). Similarly, the system trajectories in (31) reach the sliding manifold ${\tilde{x}}_{3}=0$ in finite time $ts_{2}$, depending upon the discontinuous gain ϑ. The discontinuous gain $\mu >>\vartheta ts_{1}<<ts_{2}$, hence, the sliding manifold ${\tilde{x}}_{1}=0$ is achieved much faster than the manifold ${\tilde{x}}_{3}=0$. Consequently, during the estimation of the $x_{2}$, the state $x_{4}$ is already estimated.

Now, the error dynamics of the SMO is obtained by computing the time derivative of ${\tilde{x}}_{3}$, which is given by

(32) $${\tilde{\dot{x}}}_{3}={\dot{x}}_{3}-{\hat{\dot{x}}}_{3}=k_{tl}x_{2}-\vartheta \text{sign}({\tilde{x}}_{3}).$$

The sliding mode is established if $\vartheta >k_{tl}∥x_{2}∥$, and the sliding mode equation is defined in terms of the equivalent control

$$(\vartheta \text{sign}({\tilde{x}}_{3})){\;}_{\text{eq}}=k_{tl}x_{2},$$

which can be obtained by employing a low‐pass filter, characterised by

(33) $$\begin{array}{rl} \tau {\dot{z}}_{2}+z_{2} & =\vartheta \text{sign}({\tilde{x}}_{3}),\\ \lim_{z_{2}\to 0}z_{2} & =(\vartheta \text{sign}({\tilde{x}}_{3})){\;}_{\text{eq}}. \end{array}$$

Consequently, $x_{2}$ is determined as

(34) $$x_{2}=\frac{z_{2}}{k_{tl}}.$$

It is worth mentioning that there is no need to estimate $x_{2}$ if $\dot{s}$ is obtained by a differentiator.

## 5 Results and discussions

In this section, a thorough simulation analysis for the sliding mode controller and observer pair is described for the regulation of p53 protein. Moreover, a comparison between the conventional SMC and DSMC techniques is also presented. It is worth mentioning that for a fair comparison between both techniques, the discontinuous gains (*M* and κ) are kept identical. Moreover, the challenges faced while implementation of these feedback control techniques for biological systems is catered by a rigorous simulation analysis in the presence of the practical issues.

A major challenge while developing computational models for complex biological systems is the existence of multiple free parameters. The dynamic behaviour of the model is often highly dependent upon these parameters. Although high accuracy methods for discovering interactions are well developed, accurate methods for measurement of parameters are still limited [41]. Traditionally, these parameters are estimated using regression techniques, by optimising the consensus between available data and the model.

The parameters estimated using *in‐vitro* measurements can lead to inaccuracies due to differences in *in‐vitro* and *in‐vivo* conditions. Moreover, the amount of measured data is usually limited due to expensive and time‐consuming techniques. Consequently, these approaches often yield parametric uncertainties. For the p53 model discussed in this paper [21], most of the parameters mentioned in Table 1 are constrained but the parameters $k_{f},\delta$ and γ can vary by the application of different stresses. To study the robustness property of the SMC for the p53 pathway, 20% of parametric uncertainties are introduced in the nominal parameters, as described previously in Table 2. It is worth mentioning that the controller and estimator contain the nominal system parameters.

**Fig. 4:**
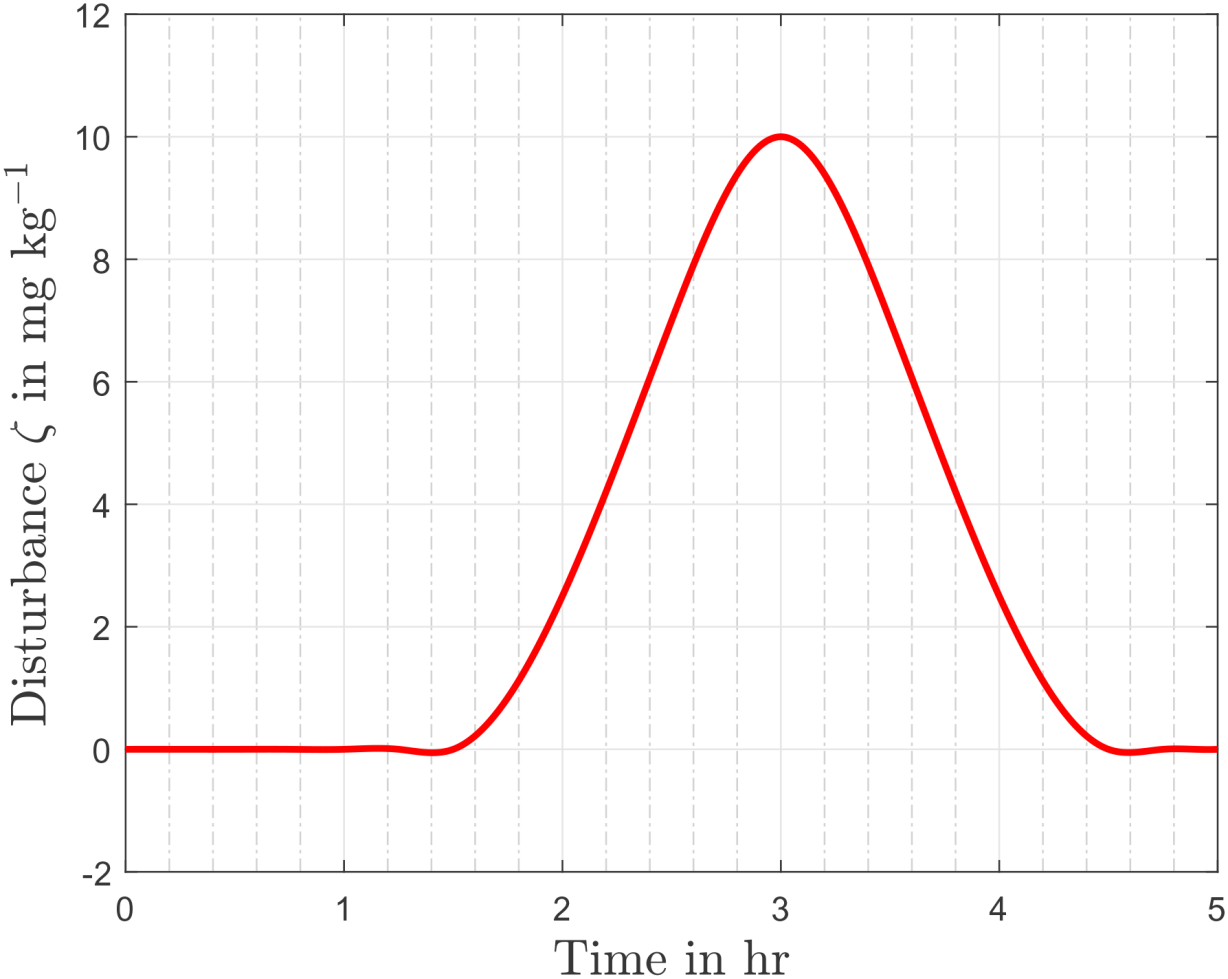
Time profile of the disturbance

A matched input disturbance ζ is also considered to ensure robustness. The amount of loss in the drug Nutlin due to unwanted cross‐talk between pathways or due to undesirable signals from neighbouring cells is considered as a disturbance. The hypothetical time profile for the vanishing disturbance is shown in Fig. 4. Moreover, the effect of measurement noise has also been incorporated. In this regard, an additive white Gaussian noise with zero mean and variance of $1\times 10^{-4}$ is added in each measurement of the p53 plant. The robustness of the proposed control scheme is assessed by introducing parametric uncertainties, external disturbance, and sensor noise simultaneously.

**Fig. 5:**
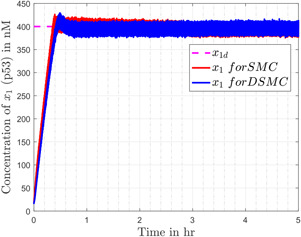
Output of the p53 pathway for both controllers

**Fig. 6:**
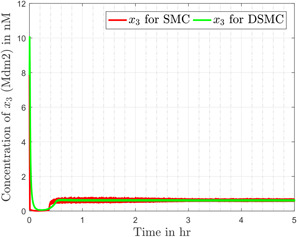
Concentration of MDM2 for both controllers

According to different studies conducted on cancerous cells in the literature, it is well noted that in normal healthy cells, the concentration of p53 $\left(x_{1}\right)$ is around 400 nM (nanomoles). In cancerous cells, p53 is prohibited from raising its level, so it remains in a lower concentration state. In the simulations $x_{1}$ is initialised for a case of the cancerous cell, i.e. 17 nM [21], and the desired concentration of p53 $\left(x_{1d}\right)$ is set to 400 nM in the controller. It is also evident from the literature that sustained p53 concentration is possible only if MDM2 concentration is kept low. The designed controller strategy ensures a sustained high level of p53 (Fig. 5) and a lower concentration of MDM2 (Fig. 6). It is evident from Fig. 5 that an excellent tracking behaviour of the output (p53) is obtained, the level of the p53 protein rises up quickly after application of the controller and maintains its desired value at steady state.

**Fig. 7:**
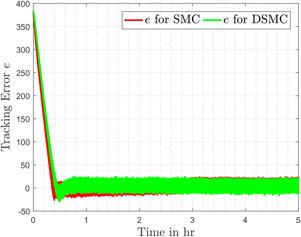
Tracking error e for SMC and DSMC

Figs. 5 and 6 compare the simulation results (for $x_{1}$ and $x_{3}$, respectively) obtained from SMC and DSMC. In Fig. 5, $x_{1}$ quickly reaches the desired value for both the cases. The continuous control introduces a small overshoot in the output and slightly increases the settling time, but that all comes with the advantage of chattering reduction in the system. The corresponding tracking error $\left(e=x_{1}-x_{1d}\right)$ in the case of SMC and DSMC is depicted in Fig. 7. Fig. 6 represents the concentration of mdm2, which is quite smooth in case of DSMC as compared to the SMC.

**Fig. 8:**
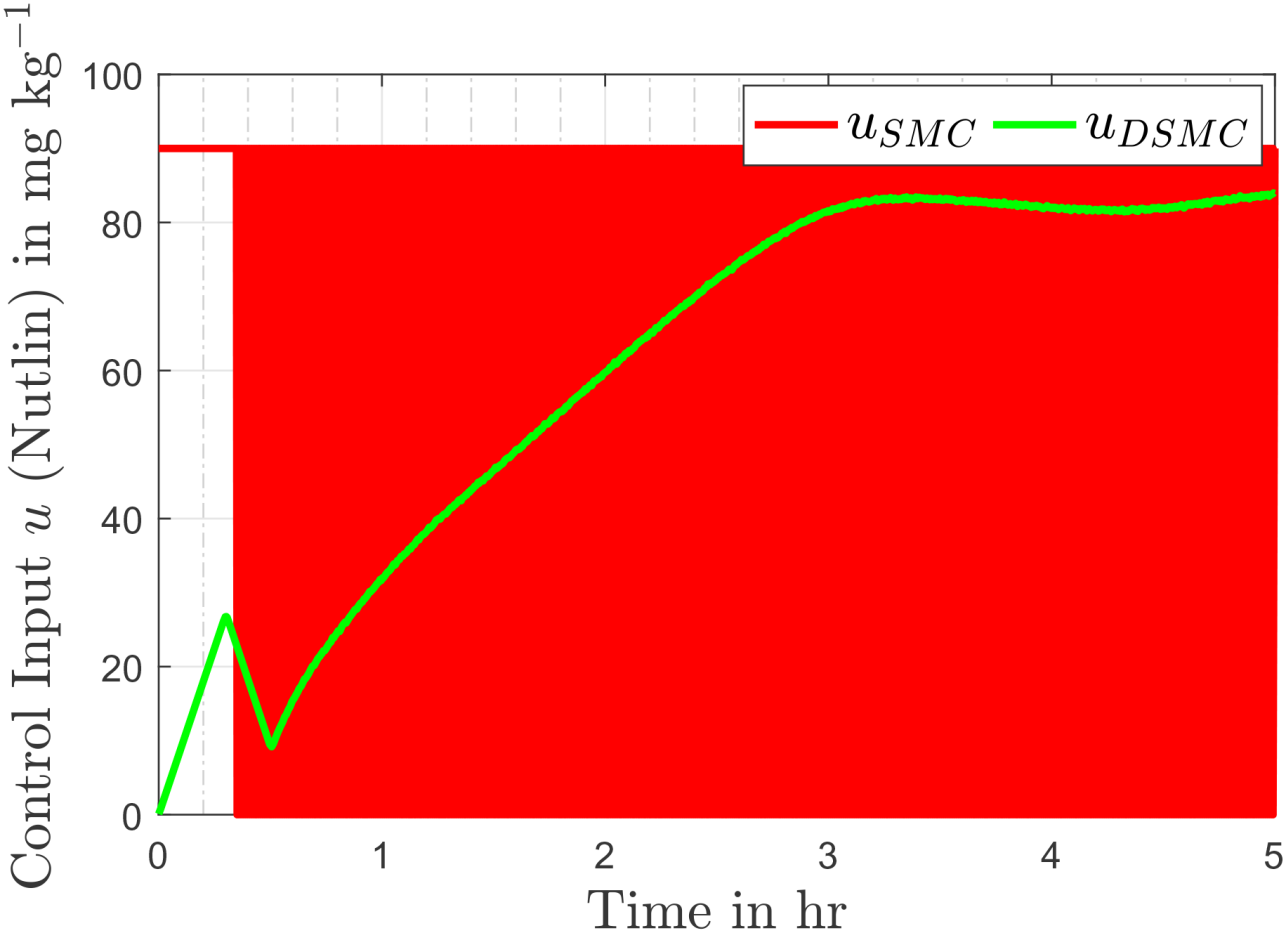
Control input (Nutlin) comparison for both controllers

**Fig. 9:**
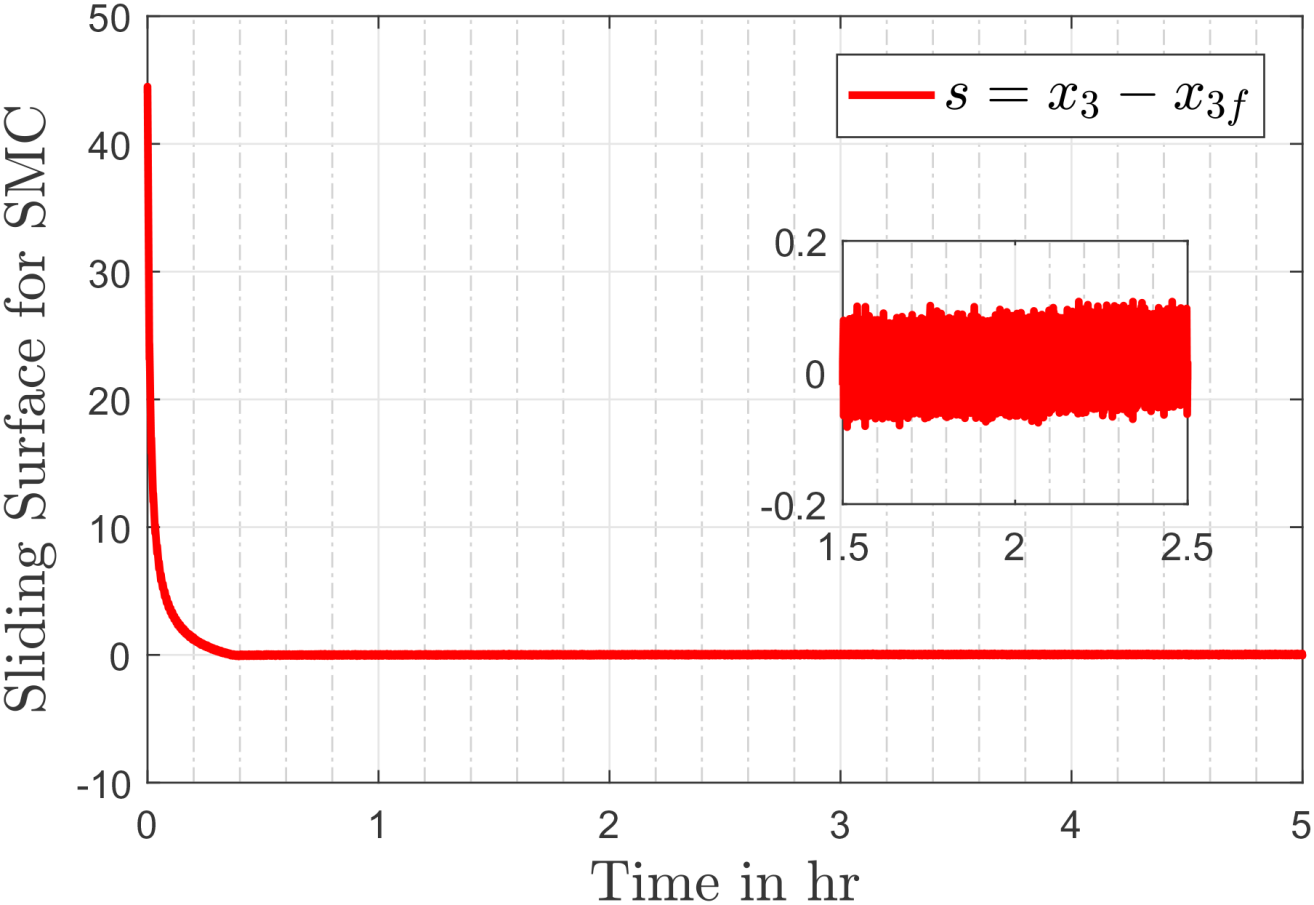
Sliding surface in the case of SMC

**Fig. 10:**
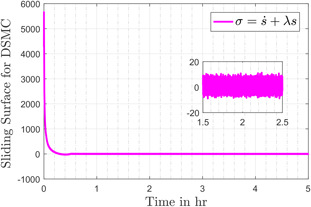
Sliding surface in the case of DSMC

Fig. 8 compares the discontinuous control input generated by first‐order SMC and the control input provided by the modified control, which is smooth as compared to its counterpart. The smoothness of input is attributed to the use of the discontinuous term in first‐order time derivative of the control input. It is evident that the control effort remains under 75 mg/kg, which is in accordance with the upper bound (i.e. 400 mg/kg) experimented by the authors in [42]. The sliding variables *s* and σ, for the conventional SMC and the DSMC, are shown in Figs. 9 and 10, respectively. In the reaching phase $\left(s\neq 0\right)$, the controller drags $x_{3}$ towards $x_{3f}$ and during the sliding motion $\left(s=0\right)$, the design of *s* keeps the tracking error *e* zero, consequently the output $x_{1}$ attains its desired value $x_{1d}$. The chattering phenomenon can also be seen in the zoomed version of Figs. 9 and 10.

A quantitative analysis is also carried out to evaluate and compare the performance of DSMC with conventional SMC. The performance criteria to measure the error, i.e. root‐mean‐square error (RMSE), is computed by

(35) $$\text{RMSE}=\sqrt{\frac{1}{N}\sum_{i=1}^{N}e^{2}(i)},\;e(i)=x_{1}(i)-x_{1d}(i),$$

where *N* is the number of total time samples. Furthermore, the average power of both control signals, defined by

(36) $$P_{\mathrm{avg}}=\frac{1}{N}\sum_{i=1}^{N}u^{2}(i)$$

evaluates the control effort efficiency. The RMSE and $P_{\mathrm{avg}}$ for both the controllers are given in Table 3. The comparison shows that conventional SMC has slightly better tracking performance than DSMC, but that comes at the cost of higher control energy consumption and discontinuous control input.

**Table 3:** *RMSE* and $P_{\mathrm{avg}}$ of different controllers

Controller	RMSE	$P_{\mathrm{avg}}$
SMC	56.3273	$6.030\times 10^{3}$
DMC	62.9805	$4.286\times 10^{3}$

## 6 Conclusion

In this paper, novel drug design is accomplished for obtaining the desired level of p53 concentration. An SMC based robust nonlinear technique for a control‐oriented model of the p53 pathway is presented for the re‐activation of wild type p53 protein. The small molecules based drug Nutlin is considered as the control input to revive p53 protein to the desired concentration level. Simulation tests are performed to evaluate the effectiveness of the control scheme, which shows promising results but with the issue of undesirable high‐frequency chattering. For smooth control actions and chattering reduction, a modified control technique based on the theory of dynamic sliding mode is presented. The modified control leads to decent trajectory tracking while guaranteeing smooth control actions. For the estimation of unmeasured system states, a reduced‐order SMO is employed. The robustness of the proposed scheme is accessed by introducing parametric uncertainties, measurement noise, and an input disturbance. Because the exact function of disturbance is unknown, a hypothetical profile is assumed. However, a disturbance estimator can be constructed in the future to better cope with the effects of the disturbance. Moreover, a quantitative comparison is also made between the DSMC and the conventional SMC, which shows that the DSMC consumes lesser control energy for the same tracking performance. The proposed control method can complement existing chemotherapy treatments and can become a valuable asset in targeted cell therapy.
